# Sex-limited chromosomes and non-reproductive traits

**DOI:** 10.1186/s12915-022-01357-5

**Published:** 2022-07-06

**Authors:** Aivars Cīrulis, Bengt Hansson, Jessica K. Abbott

**Affiliations:** Department of Biology, Lund University, 223 62 Lund, Sweden

**Keywords:** Y chromosome, W chromosome, UV chromosomes, Heterochromatin, Gene expression, Sex differences, Loss of chromosome Y

## Abstract

Sex chromosomes are typically viewed as having originated from a pair of autosomes, and differentiated as the sex-limited chromosome (e.g. Y) has degenerated by losing most genes through cessation of recombination. While often thought that degenerated sex-limited chromosomes primarily affect traits involved in sex determination and sex cell production, accumulating evidence suggests they also influence traits not sex-limited or directly involved in reproduction. Here, we provide an overview of the effects of sex-limited chromosomes on non-reproductive traits in XY, ZW or UV sex determination systems, and discuss evolutionary processes maintaining variation at sex-limited chromosomes and molecular mechanisms affecting non-reproductive traits.

## Sex chromosome systems and degeneration

**Fig. 1.**
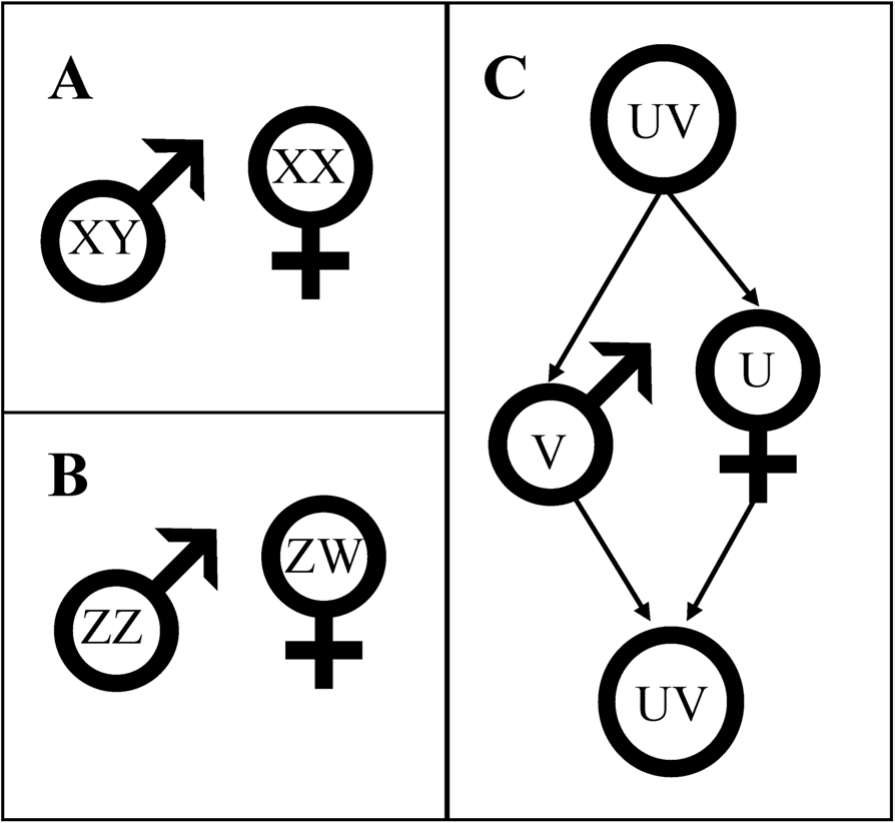
There are three sex chromosome systems. Two systems exist in diploid organisms—XY (e.g. mammals), where male is the heterogametic sex (**A**), and ZW (e.g. birds), where female is the heterogametic sex (**B**). While in haploid organisms there is the UV system (e.g. some mosses), where the female gametophyte is U and the male is V (**C**)

In species with genetic sex determination (GSD) via sex chromosomes, all differences between the sexes can ultimately be attributed to the sex chromosomes. There are three types of sex chromosome systems: XY and ZW in diploid organisms and UV in haploid organisms (Fig. 1).

In both the XY and ZW systems, one sex is homogametic (XX or ZZ) and the other is heterogametic (XY or ZW). The XY system, in which the male is heterogametic, is the most common [1] and well-known and is present in mammals, beetles, several flies and some fishes, reptiles, frogs and plants [2–4]. The female heterogametic ZW system is also widespread, being present in birds and some species of Arthropod (including crustaceans and insects), reptile (including the majority of snakes), fish, plant and amphibian [2, 4–6]. In both systems, the sex-limited sex chromosome—the chromosome found only in one sex, i.e. Y or W—has been lost in some species, resulting in XO males [7] and ZO females [8] respectively. In other species, multiple sex chromosomes have been observed, presumably due to neo-sex chromosome evolution involving autosome fusion or translocation [5]. Moreover, both types of heterogametic systems can occasionally coexist within the same species, such as in some frogs, houseflies, midges and fishes [9]. There can be several different reasons why some species evolve XY and others ZW systems. One factor is which kind of sterility mutation becomes fixed first during the evolution from hermaphroditism to separate sexes. If a dominant female-sterility mutation fixes, that drives the evolution of an XY system, but if a dominant male-sterility mutation fixes, then a ZW system evolves [7]. XY systems are more common, which could be due to stronger selection in males, which drives faster evolution of the Y [7] and makes the Z chromosome more male-biased, thus limiting female fitness and population growth, in ZW systems [9]. Similarly, deleterious mutations accumulating on the Y and decreasing male fertility are less harmful for a population compared to mutation accumulation on the W which decreases female fertility [10]. Finally, XY systems seem to provide protection from meiotic drive [7]. All of these phenomena could contribute to the observed higher frequency of XY sex determination compared to ZW sex determination. Species in clades with homomorphic sex chromosomes (e.g. fish and reptiles) can undergo frequent turnovers of the heterogametic systems, further increasing the observed diversity in sex determination.

The UV sex chromosome system is the least common variant and is found in haploid organisms: some mosses, liverworts, fungi and algae [4, 7], where U is female and V is male (Fig. 1C). In UV systems, diploid individuals are neuter, and haploid males and females are produced by meiosis. These haploid individuals then develop and grow to eventually produce gametes which fuse to produce a new diploid individual.

Common to all these sex chromosome systems is that the sex-limited sex chromosome, i.e. the Y, W, U and V chromosomes, often, but not always, undergoes sex-specific evolution, including degeneration and gene loss as a result of cessation of recombination, the causes of which are discussed below [7, 11]. The Y, W, U and V chromosomes are therefore also known as non-recombining sex chromosomes (NRSCs), which is how we refer to them throughout this review. Genes that remain functional on highly degenerated sex chromosomes are generally believed to code for sex-specific traits, such as sex cell production, and to have few or no other functions. Moreover, sex differences in shared traits have been traditionally attributed to sex hormones (at least in mammals). However, NRSCs have the potential to influence traits that are shared between the sexes, and a growing body of evidence shows that they can play a crucial developmental role as the loss or gain of an extra copy can dramatically affect the phenotype [12–14]. As the effect of these chromosomes on sex-specific traits such as sperm production is well established, it would now be valuable to switch focus to explore how these chromosomes may influence sexual dimorphism in shared traits, or even play a role in traits where sex differences are not observed. For example, it has been shown that in some cases sex hormones and sex-linked genes have opposite phenotypic effects, such that sex-linked genes reverse the effects of the sex hormones, thereby resulting in the production of an equal phenotype between males and females [15]. Thus, possible non-sexual effects of these chromosomes should not be ignored, and we show here that NRSCs can play an important role in health and disease (Table 1).

**Table 1:** Affected non-sexual phenotypes by Y chromosome in mammals

Phenotype	Mechanism of action	Species	References
*Nervous system*			
Aggression	MSY	Human	[16–20], but see [21]
	Mainly MSY acting through increased testosterone	Mouse	[22–28]
	MSY acting through increased tesosterone and decreased serotonin	Rat	[29]
Alcoholism	Y chromosome haplogroups	Human	[30]
Alzheimer’s disease	LOY	Human	[31, 32]
Anxiety	Chromosome Y consomic strains (also imprinting the daughter’s genome, thus decreasing anxiety)	Mouse	[33, 34]
Autism	Extra Y or variation in it (*SRY* and *NLGN4Y* in particular)	Human	[13, 35, 36], but see [37]
	*Sry* in interaction with the genetic background on β-endorphin levels	Mouse	[38]
Chemosensory system	MSY (chromosome Y consomic strains)	Mouse	[39, 40]
Dopamine system	*SRY* increases catecholamine synthesis and metabolism	Human	[41]
	MSY through tesosterone in hippocampus	Mouse	[42–44]
	Chromosome Y consomic strains (*Sry1*)	Rat	[45]
Hearing impairment	*DFNA49* insertion from chromosome 1	Human	[46]
Intelligence	Reduction due to extra Y, p.I679V NLGN4Y	Human	[17, 19, 21, 47]
Macrocephaly and brain size	Y chromosome increases size	Human	[14, 48]
Motor functioning	Larger or extra Y as well as *SRY* through regulation of monoamine oxidase A	Human	[13, 19, 36, 49]
	Possibly through *Sry*	Rat	[50]
Norepinephrine concentration	Possibly through *Sry*	Rat	[50, 51]
Parkinson’s disease	*SRY* as a risk factor	Human and rat	[52]
Response acquisition	Y epistatically interacts with autosome 9	Mouse	[42]
Schizophrenia	LOY	Human	[53, 54]
Stress	Y, possibly through *Sry*, interacts with other chromosomes	Rat	[50, 55]
Suicide	LOY in blood	Human	[56]
*Cardiovascular and immune system*			
Atherosclerosis	MSY (mainly lower expression of *UTY* and *Lnc-KDM5D-4*)	Human	[57–59]
Cardiomyocyte size	Due to different responses to testosterone	Mouse	[60–62]
Coronary artery disease	MSY (mainly lower expression of *UTY*)	Human	[57, 59, 63]
Hypertension	Protective role through increased expression of *BMPR2* via SRY	Human	[64]
	Y has a protective role	Mouse	[65, 66]
	Through Sry affecting several renin-angiotensin and SNS gene promoter activity	Rat	[50, 67–69], but not replicated [70]
Lipid profile	Y haplotypes (possible gene - lnc-KDM5D-4)	Human	[57, 71–73]
	Chromosome Y consomic strains	Mouse	[74]
	Chromosome Y consomic strains	Rat	[75, 76]
Na and insulin levels	Chromosome Y consomic strains (*Sry3*)	Rat	[77]
Autoimmunity	LOY in blood	Human	[78–81]
	Copy number variation of *Sly* and *Rbmy* and gain of telomeric end of the X	Mouse	[82–87]
Immune cell abnormalities	Independent of *Sry* and IFN-αβ	Mouse	[88–90]
	Loss of *CD99* on the PAR	Human	[91]
Viral infections	Haplogroup I	Human	HIV-1 [92]
	Chromosome Y consomic strains (independent of *Sry*)	Mouse	Coxsackievirus B3 [89, 93], influenza A [94]
*Other traits*			
Albuminuria	Chromosome Y consomic strains	Rat	[95]
Cancer	LOY, aneuploidy, misexpression of MSY genes in somatic cells, microdeletions	Human	[96–100]
	Loss of UTY	Mouse	[101]
	*Sry* as an oncogene	Rat	[102]
Baldness	SRY in the scalp	Human	[103]
Body size	MSY increases height independently of sex hormones	Human	[104–107]
	Y epistatically interacts with autosome 9 independently of *Sry*	Mouse	[42, 108, 109]
Diabetes	LOY	Human	[78]
Glucose metabolism	Y interacts with chromosome 2	Rat	[76]
Hirschsprung disease	SRY represses RET	Human	[110]
Hypertelorism	Extra Y	Human	[48]
Liver damage	LOY	Human	[96]
Macular degeneration	LOY in blood	Human	[111]
Mortality	Extra Y and LOY in blood decreases lifespan, while hypermethylation of Y has a protective mechanism	Human and other mammals	[78, 112–115]
Sensitivity to testosterone	MSY	Mouse	[22, 60, 62]
Tooth growth	Genes on the Yqll promote	Human	[116]

We review the literature on non-reproductive traits known (or suggested) to be affected by NRSCs. However, we believe that the list of traits is not complete, as the NRSCs may participate in other traits directly or indirectly. We also provide an overview of which mechanisms these chromosomes may influence the traits by, despite undergoing degeneration. We concentrate on all types of non-reproductive traits, except sexual behaviour and primary sex characteristics (i.e. sex-limited body structures directly involved in reproduction, such as gonads and external genitalia). We focus here on the sex-specific regions of sex chromosomes, though it should be noted that the pseudoautosomal region (PAR)—a short region of homology between sex chromosomes that behaves like an autosome and can recombine—can also play a role in sexual dimorphism. For example, there is male-biased expression in PAR genes in mammals, since one X undergoes inactivation in females [117], and in emu, since they are downregulated in females [118].

## Evolution and degeneration of sex-limited chromosomes

**Fig. 2.**
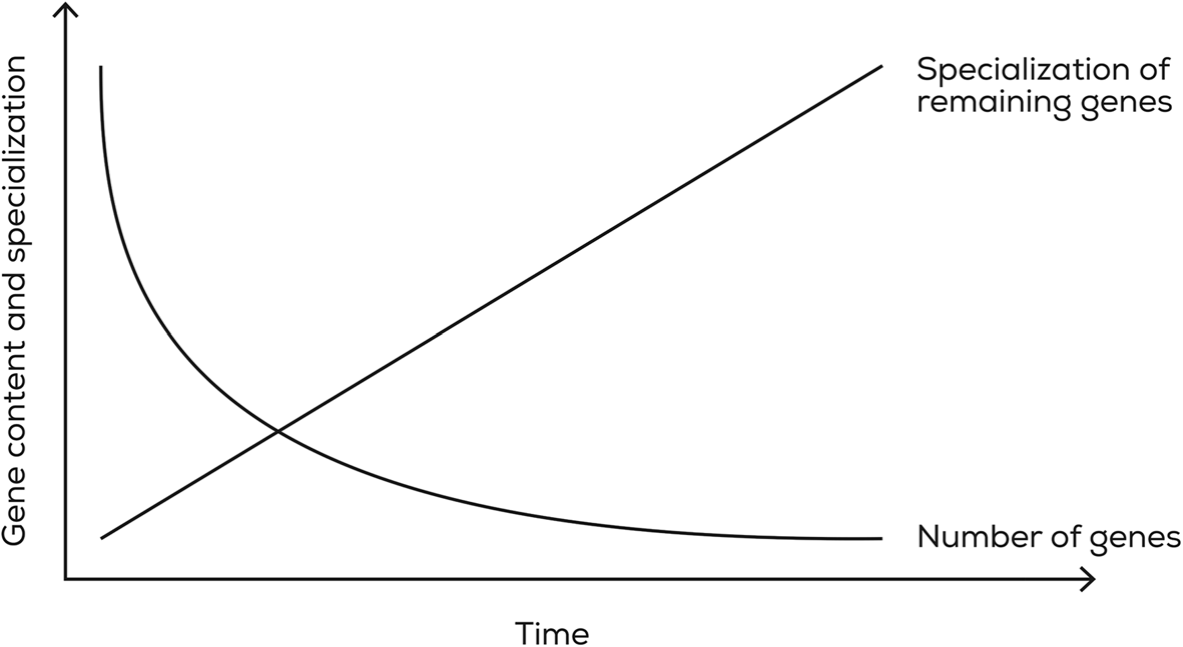
Gene loss and specialization over time on NRSCs. The rate of gene loss is expected to decrease with time since recombination cessation, as non-essential genes are lost early in this process, while essential genes can be maintained through purifying selection. In contrast, the rate of specialization may be more or less constant since it will likely be dependent on mutation accumulation

According to the canonical model, sex chromosomes start to evolve from a pair of autosomes, when one or more genes acquire a sex-determining function. Subsequently, recombination arrest may evolve around the sex-determining region and gradually expand to encompass most of the chromosome [5]. Accumulation of male-beneficial loci on the non-recombining portions of Y and V chromosomes will be favoured, as will accumulation of female-beneficial loci on the W and U. Thus, the NRSC becomes more and more specialized to code for very specific sex characteristics, while losing most of its original gene content due to lack of recombination (Fig. 2). However, not all sex chromosomes become heteromorphic (e.g. in emu [118] and pufferfish [119]). This could be due to the fact that there are different ways of resolving sexual conflicts, situations, where sexually antagonistic loci have positive effect in one sex, but negative in the other. These conflicts can be resolved either through ensuring sex-limited inheritance of sexually antagonistic genes via recombination suppression, or simply modulating expression of these genes within each sex via sex-specific transcription factors.

NRSCs (Y, W, U and V) usually contain two distinct regions: (i) the sex-specific region where recombination is suppressed, and (ii) the PAR, which serves to ensure proper segregation during meiosis [7]. Over time, the sex-specific region tends to degrade as a result of various processes, including drift, hitchhiking effects and perhaps even selection for silencing [120]. Genes that survive this degradation process are expected to have highly important “housekeeping” functions, resulting in strong purifying selection [7]. As males usually have a higher mutation rate than females and are often subject to more intense sexual selection, male-limited sex chromosomes (Y, V) are prone to faster degeneration than female-limited sex chromosomes (W, U) [7]. Although the degeneration process results in loss of functional genes over time [11, 121, 122], it does not necessarily mean that the sex-limited region inevitably decreases in physical size, as NRSCs often acquire additional genetic material in the form of transposable elements, repetitive or organellar DNA, duplications, transpositions or autosomal translocations [11, 121, 123].

Apart from low gene content, degraded NRSCs may have up to ten times smaller nucleotide diversity than autosomes [5], leading researchers to assume that they have little standing genetic variation for phenotypic traits. As discussed in more detail below, there is now good evidence that variation on NRSCs (e.g. in gene copy number or heterochromatin length [124, 125]) can have an important influence on the phenotype [126, 127], proving that low gene content need not necessarily limit the evolutionary potential of NRSCs.

## Y chromosomes

### General properties of Y chromosomes

Not surprisingly, Y chromosomes are the most studied sex-limited chromosomes as they determine sex in humans and in many popular animal model organisms. Interestingly, Y chromosomes are the most prone to degeneration (compared to W, U and V chromosomes) due to small effective population size and limitation to the male line, which is associated with stronger sexual selection and higher mutation rates (via oxidative stress, lack of repair enzymes and more divisions of sex cells) [7, 128]. This reasoning is supported by the notion that the Y chromosome seems to be lost most frequently, leading to the evolution of XO systems (e.g. in several rodent and insect species [128]). Y chromosomes are often highly differentiated and degenerated, with large amounts of repetitive sequences.

This degeneration has been implicated in longevity differences between the sexes. Cross-species comparisons have shown that the heterogametic sex has a shorter lifespan on average than the homogametic sex and that this effect is exaggerated in XY systems (>20% lifespan reduction compared to <10% in ZW systems) [112]. The exact cause is unknown, but is thought to be a result of unmasking of deleterious recessive mutations in the heterogametic sex (i.e. the unguarded X hypothesis).

### Mammal Y chromosomes

Mammal Y chromosomes are small, are highly repetitive and contain few coding genes, some of which exist in multiple copies, which increases their survival [129]. Most of the genes have testes-specific expression [5]. The mammalian Y chromosome is thought to have emerged 166 million years ago, when the sex-determining gene *SRY* arose [130]. Later on, most of the genes were lost, leaving only widely expressed and dosage-sensitive regulators of chromatin modification, transcription and splicing, translation and ubiquitination, suggesting that the surviving genes are essential and have the potential to regulate expression of target genes throughout the genome [129]. One exception to the general mammalian pattern is the platypus, which has multiple X and Y chromosomes which pair as a ring during male meiosis [131]. How this pattern has arisen is unknown, but it would be interesting to see if it results in increased recombination and gene transfer or turnover between the Xs and Ys.

The human Y chromosome is ~57 Mb large (1/3 the size of the X) and contains 64 coding and 107 non-coding genes [132]. The male-specific region of the Y chromosome (MSY) makes up 95% of the chromosome, leaving only 5% to the PAR, which occurs on both ends of the sex chromosomes (PAR1: 2.6 Mb long with ~16 genes; PAR2: 0.32 Mb long with ~5 genes) [96]. Most of the genes are ampliconic (i.e. occur in several adjacent and highly similar copies) as a result of translocation from the autosomes and Y-Y gene conversion between palindrome arms, and have testes-specific functions [133]. But several MSY genes have survived from the original ancestral autosome or have been acquired from the X or autosomes, and around half of all Y genes are expressed quite widely in the body [133, 134]. For example, two genes, *RPS4Y1* and *RPS4Y2*, code for a ribosomal protein and are homologous to *RPS4X* on the X chromosome [133]. Similarly, the gene *AMELY* is expressed in developing tooth buds and together with *TBL1Y* also in the thyroid, thus possibly coding for some kind of non-reproductive sex difference, although there are homologues on the X [133, 135].

In contrast to other mammalian Y chromosomes, the mouse Y is almost entirely euchromatic except for the centromeric region and contains relatively many coding (172) and non-coding (570) genes [132]. Although it has lost more of its original genes than the human Y, the mouse Y chromosome has managed to acquire many newer testes-specific genes from autosomes due to a history of meiotic drive initiated by the X, and the similarity between the primate and mouse MSY is only 2.2% [136]. Four mouse Y chromosome genes are widely expressed throughout the body (*Ddx3y*, *Eif2s3y*, *Kdm5d* and *Uty*), but it is not clear whether these genes play a role in sex differences since they all have X-linked homologues [136].

The main function of the mammal Y chromosome depends only on a single gene—the male-determining *SRY*, which is a transcription factor controlling expression of numerous genes in a sex-specific manner. SRY activates another transcription factor gene, *SOX9*, which represses ovarian genes and activates testicular genes, determining Sertoli cell fate, thus continuing formation and maintenance of the male gonad. This eventually leads to testosterone production after the testes are fully formed. Sex hormones then act on different tissues throughout the body to produce secondary sex differences. Mammal sex determination can therefore be seen as a two-step process, where the sex chromosome content determines the fate of bipotential gonadal ridges to develop into testes or ovaries. Then these genetically determined gonads start to produce sex hormones, which in turn drive the sexual differentiation of the body (i.e. phenotypic sex) [137]. Although it has traditionally been accepted that sex hormones masculinize or feminize the body [138], during the last 30 years evidence has accumulated that direct sex chromosome effects also help to establish sex differences independently of sex hormones. During the lifespan of males, the magnitude of the effects of the Y and testosterone changes, with the Y being the most important in the very beginning of the pregnancy until start of testosterone production, and then again in the end of life, when testosterone levels drop dramatically [139].

**Fig. 3.**
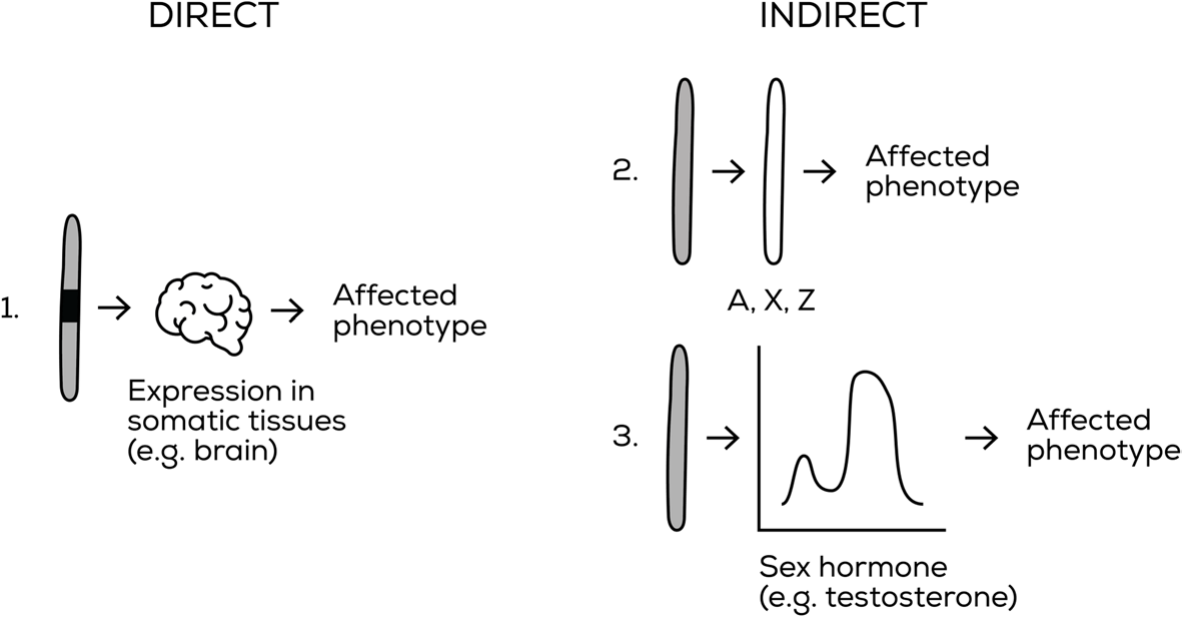
NRSC (in grey) mechanisms of action (Table 1). Direct mechanisms include (1) protein-coding gene expression in somatic tissues, while indirect mechanisms are exerted through (2) regulation of other chromosomes (via transcription factors, non-coding RNAs, heterochromatin effects or imprinting) or (3) sex hormones

As a result of our survey of the literature, we have identified three main mechanisms by which the Y chromosome can affect somatic traits in mammals, one of which is direct and two are indirect:

1. Changes in amino acid sequence or expression level of Y-linked protein-coding genes expressed in somatic tissues (direct) (Fig. 3). Allelic variation which affects expression could occur via mutations in coding regions, promoters or regulatory regions. Because of the heterochromatic nature of the Y, expression of Y-linked genes could also be influenced by consistent differences in heterochromatin distribution between haplotypes.
2. Y chromosome modulation of expression of X-linked, autosomal or Y-linked protein-coding genes (Fig. 3), for example via transcription factors, non-coding RNA genes, or heterochromatin effects (indirect). Transgenerational effects of the paternal Y on daughters (see below) would also fall into this category.
3. Modulation by testosterone (indirect) (Fig. 3). Variation in Y-linked genes may result in variation in testosterone levels and associated receptors. This will have many carry-over effects on the phenotype, which are indirectly attributable to Y-linked genetic variation.

These mechanisms are potentially applicable to any sex-determining system and are not exclusive to mammals. Since genes occur in interaction networks, these categories are not mutually exclusive either, and in practice, it can often be difficult to distinguish, e.g. effects mediated by testosterone from direct effects of the Y genotype. However, we feel that it is worthwhile to make a distinction between categories since the evolutionary dynamics of each type of mechanism may differ. For example, regulatory effects of Y genes are likely to be more evolutionarily labile than protein coding changes [140]. Since humans and other mammals are so well studied, they are well suited for providing a framework which can be extended to other species. Below, we first discuss the limitations of various methods of detection of the effects of NRSCs, and then move on to discussing each main mechanism of action in turn.

#### Methods of detection of effects of NRSCs

Which methods are used for detecting effects of NRSCs place limits on our ability to determine the mechanism of the effect (direct, indirect or primarily hormonal). Before the advent of modern methods of studying gene expression, Y chromosome copy number could be used as a proxy for expression differences in Y-linked genes, for example via natural variation in Y chromosome number via sex chromosome aneuploidies [12–14, 21, 141, 142] or somatic loss of the Y chromosome via aging [78, 96]. Although this method does not require modern sequencing technology, it cannot distinguish between changes in expression of protein-coding genes (category 1) versus regulatory effects (category 2), since the Y chromosome encompasses both types of genes. However, it could be possible to exclude hormonal effects (category 3) if testosterone level does not differ between individuals with different numbers of Y copies. Another simple method for detecting effects of Y-linked genes is through correlations between Y haplotype and phenotypic differences [143]. Again, without more detailed information about differences between haplotypes, it is difficult to know if differences are mainly due to direct or indirect effects, but it can be possible to exclude hormonal effects as the cause, if testosterone levels do not differ between haplotypes [144].

Expression of Y-linked genes in somatic tissues can be measured directly using standard methods such as microarrays or RNA-seq [145]. For example, seven Y genes have been found to be widely expressed in different organs in mice [136]. Depending on how well the functions of the differentially expressed genes are known, it can then be possible to assign effects to category 1 (mainly driven by protein-coding genes) or category 2 (mainly driven by regulatory effects). Similarly, RNA-seq can be useful for detecting effects of non-coding RNAs [146]. However, it is important to note that expression of Y-linked genes in somatic tissues in adults could potentially be influenced by circulating testosterone, as it has been shown that testosterone affects methylome and transcriptome of the genome [147]. These effects therefore always need to be considered, and if possible, controlled for.

The highly degenerated nature of the Y chromosome has previously made it difficult to sequence, but long-read methods have helped to overcome these issues [132]. This means that GWAS can be used to detect allelic differences in protein-coding or regulatory genes that are associated with phenotypic differences also in situations where the Y chromosome has been traditionally excluded. Proteomics could also be used for detecting protein-coding changes in Y-linked genes that affect the phenotype, although we did not discover any examples of this in our survey of the literature. Similarly, ChIP-seq (chromatin immunoprecipitation with massively parallel DNA sequencing) could be used to identify effects of Y heterochromatin conformation on the phenotype. This method has not been used for Y-linked genes in mammals to our knowledge, but has been successfully applied in *Drosophila* [148]. There is clearly scope for increased use of modern genomic methods to detect effects of NRSCs on non-sexual traits.

#### Y-linked protein-coding gene expression in somatic tissues

Several Y chromosome genes are expressed in pluripotent stem cells in vitro [146, 149], and embryonic rodent neurons (XX vs. XY) in cell cultures can undergo sexual differentiation even if there are no sex hormones present. Moreover, rodent dopaminergic neurons sustain genetically induced sex differences even after sex hormone or their inhibitor treatments in vitro; however, it is not known, if the effects are caused by the Y [150, 151]. Studies on humans with sex chromosome abnormalities (XX vs. XY vs. XXX vs. XXY vs. XYY vs. XXYY vs. XXXXY) clearly show that Y chromosome number has a significant effect on neurobiological phenotypes [12–14, 21, 141, 142]. For example, it seems that the Y increases brain size, possibly affecting language, emotions and other phenotypes [14]. However, as noted above, these differences are likely driven by changes in expression of both protein-coding and regulatory genes.

Other indirect evidence of an important role of expression of Y-linked protein-coding genes in somatic tissues is the association of lifetime-acquired loss-of-Y (LOY), which shows racial differences and is associated with several diseases and overall mortality [78, 96]. Surprisingly, in the case of leukaemia, LOY actually has a protective effect [97], which is very unexpected given that the protein-coding *UTY* gene on the Y acts as a tumour suppressor [101]. LOY is the most common somatic mutation in males and occurs through centromere dysfunction or telomere attrition; it increases with age and is accelerated by smoking and outdoor air pollution, alcohol, obesity and exposure to insecticides and polycyclic aromatic hydrocarbons [96]. Although these multifarious associations of diseases with LOY are intriguing, it must be noted that it is currently unclear whether LOY is causal in most cases. It is also unclear whether changes associated with LOY are mainly driven by the loss of protein-coding or regulatory genes. However, there is evidence that both are possible, since LOY has a direct effect on autosomal dysregulation in immune cells [152] and leads to decreased CD99 immunoprotein on leukocyte surfaces, because the *CD99* gene from the PAR of the Y is lost [91].

Few studies have directly quantified how Y-linked expression variation in protein-coding genes influences somatic traits. However, there is a relatively large literature investigating how Y chromosome identity (chromosome Y consomic rodent strains or haplotypes in humans) can affect various phenotypes. For example, Y chromosome identity can affect blastocyst cell number [153] and male embryo weight [108] as well as adult body size [42, 109, 154] independent of gonadal hormones in mice. Meanwhile, vulnerability to alcohol dependence [30] and autism [35] is affected by Y chromosome haplotype in men; however, here it is impossible to disentangle direct effects of differences in expression of Y-linked genes from indirect effects of testosterone, since possible differences in testosterone levels between the haplogroups are not controlled for. It is also not clear if all of these differences in mammals are driven by different Y gene expression or other effects such as Y heterochromatin silencing genes on nearby chromosomes. Nevertheless, at least some of the variation is likely attributable to allelic differences resulting in changes in the expression of Y-linked protein-coding genes.

The best evidence of direct effects of Y-linked gene expression on phenotypic differences is in coronary artery disease, where it has been found that haplogroup I has ~50% higher age-adjusted risk than eight other haplogroups. The effect seems to be driven by downregulation of two Y chromosome genes, *UTY* and *PRKY* (both of which are protein-coding), and not by traditional cardiovascular risk factors [143]. Interestingly, it is known that the difference is not driven by steroid hormonal effects as there is no difference between the haplogroups in these traits [144].

#### Y-mediated regulatory effects

There is good evidence that the Y chromosome can regulate the expression of genes on other chromosomes. For example, it has been shown that male embryonic stem cells have a unique transcriptome profile, in which the Y chromosome affects expression of 294 genes in mice [149]. These early regulatory effects may have important consequences for the later development of sex differences in different organs.

*SRY* is of course one of the most important regulatory genes on the Y, and it is known to be differently expressed in the brain based on the Y chromosome it resides on [50]. Another study has also found that *SRY* expression in human cell cultures and rats can be linked to Parkinson’s disease [52]. Thus it seems that *SRY* participates in sexual differentiation of the brain [155, 156]. Experimental allergic encephalomyelitis and myocarditis in chromosome Y consomic mouse strains may be caused by the natural variation in copy number of the *SLY* and *Rbmy* genes, which affect expression of genes in immune cells, such that the higher the copy number the lower the expression in immune cells [82].

In some cases, the specific regulatory pathways are known. In midbrain dopamine neurons, *SRY* positively regulates catecholamine synthesis and metabolism, possibly explaining male bias in fight-flight behaviours and “dopamine disorders”, such as Parkinson’s disease and schizophrenia [41]. *SRY* also directly regulates expression of the *monoamine oxidase A* gene located on the X, possibly explaining sex differences in attention deficit hyperactivity disorder, depression and autism [36]. Finally, *SRY* can be expressed in the colon of Hirschsprung’s disease patients and explain the 5:1 male bias by repressing expression of *tyrosine kinase receptor RET*, a gene responsible for almost half of the cases [110].

It is also known that the Y chromosome can regulate gene expression through other epigenetic mechanisms. For example, *SRY* and *SLY*, which are present in multiple copies, can regulate chromatin structure beyond the Y [157, 158], and the Y chromosome-linked long non-coding RNA (lncRNA) lnc-KDM5D-4 decreases expression of *PLIN2* located on chromosome 9 [57]. *PLIN2* is involved in lipid droplet formation in hepatocytes, thus possibly protecting from fatty liver, which in turn could protect from atherosclerosis and coronary artery disease. Moreover, this lncRNA is expressed across the body and may therefore account for other discrepancies among men and women in health and disease [57]. The Y chromosome also regulates genes elsewhere in the genome through small RNAs [94].

It is worth noting that indirect intergenerational effects of the Y are also possible. The Y chromosome codes for minor histocompatibility antigens against which females can create an immune response. This immune reaction could in turn result in obstetric and neonatal complications, preterm birth or lower birth weight, and stillbirth or miscarriages, leading to a female-biased sex ratio in subsequent children [159]. Moreover, mothers seem to develop antigens to extracellular NLGN4Y (a growth factor) during each male pregnancy with additive effects, which may result in feminization of the male embryos’ brain and increase the chance that the individual will be homosexual [160]. The Y chromosome also plays a role in different transgenerational effects in mice, affecting several traits in daughters by epigenetically imprinting other chromosomes [83].

#### Testosterone-mediated effects of the Y chromosome

Testosterone affects many traits and has traditionally been considered the main way the Y can play a role in somatic phenotypes. Consistent with this, chromosome Y consomic mice strains have indeed shown that the Y can affect testosterone sensitivity [22, 60]. A few examples of testosterone-mediated traits where MSY identity is known to be important include discriminability of individual urine odours and serotonin levels in rodent chromosome Y consomic strains, influencing aggression [23, 24, 29]. In consomic mice strains, the Y identity also affects the size of cardiomyocytes [61] due to different responses to postpubertal testosterone [60].

From these examples, it is clear that the Y chromosome has the potential to affect a wide variety of somatic traits in multiple organ systems in mammals. In many cases, an effect of the Y can be detected, but it is not always possible to determine by which mechanism. As mentioned above, in studies that have found phenotypic variation associated with different Y haplotypes (Table 1), it is unclear whether this variation is a result of differences in Y-linked gene expression, regulatory effects, variation in testosterone production, or (most likely) some combination of these. Increased use of modern genomic and proteomic methods should help to disentangle these various mechanisms in future.

### *Drosophila* Y chromosomes

*Drosophila* Y chromosomes completely lack recombination, as recombination is entirely absent in males. The Y chromosome of *D. melanogaster* is the best-studied and is at least 60 million years old [7]. It is almost entirely heterochromatic and contains only around 20 protein-coding genes gained from autosomes [161], while being roughly the same size as the X [162]. In fact, the current rate of gene acquisition on the *Drosophila* Y is eleven times higher than gene loss, so that gene content is actually increasing [163]. As many Y-linked genes in *Drosophila* have counterparts on autosomes, it is not clear if they have a male-specific function or are simply redundant [164]. However, a study of 22 Diptera species showed that most genes on old Y chromosomes have been hijacked from autosomes and then have undergone convergent evolution acquiring male-specific functions [165], suggesting that translocation to the Y may often be associated with the evolution of male-specific functions.

The protein-coding genes on the *Drosophila* Y are expressed only in the testes. The rest of the Y consists of two RNA-coding genes (the *bobbed* and *crystal* loci), long satellite DNA repeats and transposable elements [166]. The rDNA locus is the only shared one between the X and Y—no other homologues occur [165]. Dosage compensation (by male upregulation of the single X) seems to have evolved very early in the evolutionary history of *Drosophila*, which may be the reason why the Y degenerated so quickly [7]. Contrary to mammals, the Y chromosome does not have a sex-determining function in this group. However, it is important for male fertility as XO males are sterile [166].

Of the four potential mechanisms of action that we identified in mammals, only the second one (Y chromosome regulatory effects) seems likely to play an important role in *Drosophila*. Since Y-linked protein-coding genes in this species are almost exclusively related to sperm production and are limited in their expression to the testes, direct effects of Y-linked protein-coding genes via somatic expression (category 1) are unlikely, at least for non-sexual traits [166]. Sexual differentiation in *Drosophila* is controlled by the gene *doublesex*, which is alternatively spliced in males and females [7]. This means that, in contrast to mammals and birds, sex hormones do not seem to play a major role in sexual differentiation in this species [167], which suggests that hormonal mediation by Y-linked loci (category 3) is unlikely as well. We might therefore predict a priori that Y chromosome effects should mainly occur via non-coding regulatory effects in *Drosophila*, e.g. through small non-coding RNAs or heterochromatin effects [166].

Indeed, it has been shown that the Y chromosome may influence the expression of up to two thousand genes located on other chromosomes [168], depending on the genetic background and apparently mediated by heterochromatin formation, affecting immunity and olfaction [169]. Later results confirm that the Y chromosome seems to act as a “heterochromatin sink”—i.e. that the cell can only produce a certain amount of heterochromatin-inducing factors and that the Y chromosome seems to preferentially sequester these factors, hindering heterochromatization of repetitive regions on other chromosomes [148]. It has been shown that variation among Y chromosomes in their repetitive DNA content generates different patterns of chromatization and thus gene expression across the rest of the genome [170]. Nevertheless, it is worth bearing in mind that although the heterochromatin sink model of Y-linked regulatory variation currently has the best support, other mechanisms have not yet been ruled out.

Traits that have been shown to be influenced by the Y in *D. melanogaster* include longevity [162, 171], abdominal bristle number and geotaxis [172], immune gene regulation [173], same-sex sexual behaviour in males [174] and others [169, 175]. Interestingly, the paternally inherited Y chromosome and the maternally inherited mitochondria affect locomotive activity, although there is no support for any interactions between the Y and mtDNA [176]. Moreover, the Y chromosome may also affect daughters’ egg-to-adult survival rates, although the exact mechanism behind this effect is unknown [177, 178].

### Y chromosomes in other species

Although non-sexual effects of the Y chromosome are best documented in model organisms, information from other taxa is increasing. For example, in several fish and insect species colouration, genes have become linked to the sex-determining locus, presumably as a way of mitigating sexual antagonism [7].

There is also evidence of Y-linked modulation of autosomal colour genes in guppies (*Poecilia reticulata*) [179]. And several other species of live bearing fish have alternative reproductive tactics (e.g. “courters” and “sneakers”) that have been shown to be controlled by Y-linked loci [180, 181]. These alternative morphs often differ in a suite of traits, including behaviour, colouration, cognition, life history, body size and morphology [180, 182], consistent with widespread regulatory effects of Y-linked loci.

Interestingly, a recent study in *Callosobruchus* seed beetles revealed that Y-linked genetic variation could explain the bulk of the response to artificial selection on body size in lines that were selected for increased sexual dimorphism [183]. This was a surprising finding since body size is a trait that is usually expected to be controlled by many small-effect autosomal loci, but is consistent with the results from live bearing fishes discussed above.

## W chromosomes

### General properties of W chromosomes

ZW (female heterogametic) systems are also widespread and in many aspects resemble XY systems (Fig. 1B). As mentioned above, comparative studies show that ZW systems are associated with greater longevity in males, suggesting that there may be deleterious effects of the W [112]. Similar to Y chromosomes, W chromosomes are expected to have low genetic diversity due to low effective population sizes [7], although this effect may be less exacerbated in W chromosomes since females usually have lower variance in reproductive success than males [184]. One major difference between the W and the Y is, however, the maternal co-inheritance of the W and mitochondria, which may introduce cyto-nuclear associations [185]. So far there is little data available to test for evidence of coevolution of the W and mitochondria.

### Avian W chromosomes

Most data on W chromosomes come from birds. The avian ZW chromosome system is around 140 million years old, and the Z and W are highly heteromorphic. The W has lost most of its genes in most species, with the exception of some palaeognaths (e.g. ostrich) which have a large PAR [186]. Analogous to the mammalian Y, the avian W chromosome is basically a degraded counterpart of the Z chromosome. In *Ficedula* flycatchers, a passerine bird genus, 46 W-linked genes have been detected [187]. In chicken, the most well-studied bird species, 28 intact W genes are found [188], and all are single-copy except *HINTW*, which is present in multiple copies and appears to have been subject to positive selection [189, 190]. Neither the chicken W nor the flycatcher W has known acquired genes, and data from other birds or/and other independently evolved lineages (e.g. snakes) are needed to show whether this is a general feature of female-specific chromosomes. None of the 28 chicken W genes are expressed exclusively in female-specific tissues [188], and all 27 single-copy Z-W pairs are expressed in the developing chicken blastoderm, which means that the combined expression of the Z-W gene pairs in females is comparable to the expression of the two Z homologues in males [191]. Thus, the few remaining single-copy chicken W genes’ main function could be to ensure female survival by providing correct dosage (birds lack chromosome-wide dosage compensation mechanisms in contrast to mammals [192]), especially for those functioning in critical signaling pathways during early embryonic development.

It has been suggested that the relative simplicity of the W chromosome, with only broadly expressed ancestral genes and only one multicopy gene family, may be because its transmission is restricted to the female germ line. In contrast, X, Y and Z chromosomes pass through the male germ line, and all have acquired and amplified testis-expressed gene families [193]. The marked absence of acquired genes that are specifically expressed in the ovary or other female-specific tissues, even on a female-specific chromosome, suggests that, at least in amniotes, there is greater pressure to preserve or enhance male reproductive functions [193].

All three of the main mechanisms of action identified for mammalian Y chromosomes above seem plausible for avian W chromosomes as well. Because all functional W-linked genes seem to be broadly expressed, variation in amino acid sequence or expression levels of protein-coding genes (category 1) in somatic tissues could have widespread effects [194]. W-linked modulation of expression of other genes (category 2) is also possible. For example, *HINTW* is a truncated counterpart of the Z-linked *HINTZ*, and its gene product has been suggested to act as a dominant negative version, blocking a possible testis-specific function of *HINTZ*. Evidence for this function is however limited, as misexpression of *HINTW* does not disturb male gonadal development in chicken, zebra finch and emu [188]. Finally, the W could also potentially influence non-sexual traits via hormonal effects (category 3), although this mechanism may be of lesser importance in birds than in mammals. Evidence of cell-autonomous sex determination in chickens has emerged from the study of lateral gynandromorphs [194], along with sexually dimorphic gene expression that precedes gonadal differentiation [191, 194], suggesting that many sex differences are established independently of the action of sex hormones in birds. In addition, it is currently unclear whether W-linked genes have important effects on sex steroid levels in birds.

There are currently few examples of W-linked effects on non-sexual traits in birds. Genetic female (ZW) zebra finches (*Taeniopygia guttata*) with testes develop a feminized song system [195], suggesting that some sort of direct effect of the sex chromosomes determines this trait [196]. Apart from broad expression in the developing embryo, two W-linked genes (*CHD1W* and *ASW*) have been shown to be expressed in the adult brain, indicating possible but unknown functional roles [197]. Several colour pattern traits also seemed to be influenced by the W chromosome, including zebra finch beak colouration [198], blue egg colour in the common cuckoo (*Cuculus canorus*) [199] and eggshell patterning in the great tit (*Parus major*) [200].

### W chromosomes in other species

Most Lepidoptera have a pair of differentiated ZW sex chromosomes. However, in contrast to the other systems we have discussed so far, lepidopteran W chromosomes are thought to have been acquired secondarily [201]. This is because in most lineages outside of the division Ditrysia (which comprises 98% of all species), as well as in the sister order Trichoptera, females lack a W chromosome. Pronounced heterochromatization and transposable element content suggest that lepidopteran W chromosomes consist largely or entirely of repetitive sequences [202]. Accordingly, the total number of coding sequences found on the lepidopteran W is extremely low, with little overlap between distantly related species [203–205]. Some families also seem to have experienced a secondary loss of the W [206], suggesting that the W chromosome is dispensable for the genome in some species, which is consistent with its heterochromatic nature and scarcity of genes [207, 208]. We can therefore speculate that, as in *Drosophila*, the most likely mechanism of action of lepidopteran W chromosomes is a heterochromatin “sink”. To our knowledge, no non-sexual effects of lepidopteran W chromosomes have been reported to date.

W chromosomes in other systems, including fishes, frogs and reptiles, are generally poorly studied and seem in many cases to escape extensive degeneration via either environmental sex reversal and subsequent recombination between Z and W chromosomes, or frequent turnovers [7], placing them outside the scope of this review. However, similar to the livebearers discussed above, female-benefit coloration seems to be affected by the W in cichlids [209]. In addition, snakes with heteromorphic ZW sex chromosomes generally seem to lack chromosome-wide dosage compensation [210]. This suggests that remaining W-linked genes could potentially have important effects on early embryonic development, as in birds.

## U and V sex chromosomes

U and V sex chromosomes are found in organisms with haploid GSD [4, 7]. As mentioned in the introduction, cells from neuter diploid individuals undergo meiosis to produce haploid U-bearing female and V-bearing male gametophytes (Fig. 1C). U and V chromosomes have a larger effective population size (50%) relative to the autosomes compared to Y and W chromosomes (25%), since they are present in every second haploid individual (assuming equal sex ratios). Since they typically occur in species with a well-developed haploid life stage, they experience purifying selection on deleterious recessive mutations to a greater extent than Y and W. These sex chromosomes are therefore not prone to degenerate via gene loss, but rather tend to differentiate via chromosomal rearrangements (such as inversions and translocations), and accumulate sex-specific genes and transposable elements, leading to lower gene density relative to the autosomes [7]. The female U chromosome is usually larger, which is perhaps to be expected if the male-limited V chromosome is prone to faster degeneration due to higher mutation rates and more intense sexual selection in males [7].

UV sex chromosomes are the least studied of all the NRSCs and have only been well-characterized in a handful of species. In the liverwort *Marchantia polymorpha* the U and V chromosomes are highly diverged, despite the fact the males and females are phenotypically almost monomorphic [211, 212]. The male-specific V chromosome contains high amounts of repetitive DNA and two unique genes *ORF162* and *M2D3.5* [211]. In brown alga *Ectocarpus* sp., the sex-determining regions ceased to recombine more than 100 million years ago and there is evidence that they are now evolving rapidly [213]. Both the U and V chromosomes are similar in size and structure and moderately degenerated, containing ~20 genes with relatively low expression [213]. Interestingly, in this species, the PAR is enriched in transposable elements and has a low gene density [213], which is a pattern that is not typically seen in Y and W chromosomes. This species also exhibits a low level of sexual dimorphism [213]. Finally, the U and V sex chromosomes of the moss *Ceratodon purpureus* were recently characterized, revealing rather low levels of degeneration (mainly in the form of increased number of transposable elements) since their origin around 300 million years ago [214]. This suggests that recombination cessation is not sufficient in itself to drive gene loss on NRSCs.

As with W chromosomes, all three mechanisms of action are plausible in UV systems, although the importance of hormonal effects is unclear since plants (which comprise the majority of UV systems) do not have specialized masculinizing and feminizing sex hormones (even though various hormones may play an important role in sexual development) [215, 216]. Because U and V chromosomes are less prone to suffer gene loss, it seems likely that coding sequence differences and variation in somatic expression could have important effects on non-sexual traits, either directly (category 1) or indirectly (e.g. via modulation of expression of autosomal genes; category 2). However, given the low levels of sexual dimorphism in many species with UV sex chromosomes, it is unclear what traits might be affected. Nevertheless, results from *C. purpureus* are consistent with widespread non-sexual effects of U and V chromosomes. Using a quantitative genetic approach, significant sexual dimorphism and additive genetic variance for total mass and leaf length has been found and that male and female juvenile growth were not genetically correlated [217]. These differences were presumably driven by sex-linked loci. Similarly, another study [214] showed that >1700 U- and V-linked genes were widely expressed in somatic tissues in *C. purpureus*, which is substantially larger than the number of autosomal genes with sex-biased expression, suggesting that direct effects are likely to be more important than indirect regulatory effects in this species. Although genes with conserved reproductive functions were enriched among the U- and V-linked genes with somatic expression, it seems unlikely that none of these genes would affect non-sexual traits as well. There is clearly scope for further research in this area.

## Conclusions and perspectives

Although mammal Y chromosomes are gene-poor, it is clear from results in *Drosophila* and UV systems that cessation of recombination leads to differentation, but not always to the inevitable loss of genetic material. Various processes have been linked to the maintenance of coding and non-coding variation on NRSCs, including sex reversals which allow recombination between the X and Y (or Z and W), Y recombination at palindromic sites, X-Y transposition [7], purifying selection on essential genes [218, 219], translocation from the autosomes and gene conversion [220], and duplication [221]. It is therefore far from obvious that NRSCs are an evolutionary “dead end”, and our survey of the literature clearly shows that a dearth of protein-coding genes does not necessarily mean that these chromosomes have no evolutionary or genetic potential.

Moving forward, it should be possible to use knowledge of the biology of a given species to predict by which mechanisms the NRSC could affect non-sexual traits. For example, in species with a similar biology to *Drosophila*—such as a lack of sex hormones and few coding genes on the Y/W/U/V—the NRSC should mainly act through epigenetic or regulatory effects. Such an approach would enable more focused and systematic detection of wide-ranging effects of NRSCs.

Our survey of the literature has revealed that almost any type of trait may be influenced by NRSC, making it challenging to find any commonalities. This is likely to some extent due to the haphazard nature of research in this area to date, but arguably also an inevitable product of the wide range of taxa that have been studied. Nevertheless, phylogenetic comparisons suggest clear links between the NRSC and longevity in diploid systems [112], and there is scope for further research in this area. For example, is there a relationship between sex differences in longevity and the relative size of the sex chromosomes compared to the rest of the genome? Or are sex differences in longevity better explained by the relative size of the non-recombining region compared to the PAR? Do ZW species experience a mosaic loss of W (LOW) similar to the LOY that has been observed in humans, and does this contribute to decreasing fitness with age or decreased longevity in females? This question was recently addressed in two long-lived bird species, where researchers did not find evidence for LOW [222]. However, we argue that the phenomenon of LOY and LOW could be found in other species. As the LOY has been shown to be accelerated by smoking [223], we suggest that LOW in birds, for example, might be detectable in urban birds, which experience toxins similar to smoking.

More speculatively, could NRSCs contribute to the genome-wide resolution of sexual antagonism, since they seem to often have extensive regulatory effects? Sexually antagonistic alleles are those which are beneficial in one sex, but deleterious in the other [224]. In the canonical model of sex chromosome evolution, it is the existence of sexually antagonistic loci in the PAR which favours the evolution of recombination cessation, in order to ensure that male-beneficial alleles are inherited together with the male-determining region, and female-beneficial alleles together with the female-determining region [7]. However, it is also possible that sex-limited chromosomes may acquire genetic variation which helps to resolve sexual antagonism post-recombination cessation [225]. Our survey of the literature found that a number of traits which have been previously associated with sexually antagonistic selection pressures can be influenced by the NRSC. For example, cholesterol levels and height in humans [226], colour genes in live bearing fish [7], same-sex sexual behaviour in *Drosophila* [174], and body size in *Callosobruchus* seed beetles [227] are all traits which have been previously shown to be sexually antagonistic, and which here were found to be affected by Y genotype. This provides some intriguing first evidence that NRSCs may play a more important role in resolving sexual antagonism than has previously been appreciated, even when highly degenerated.

Finally, we would like to highlight the unique potential of the currently understudied UV systems. In these systems, it is possible to disentangle effects of cessation of recombination from hemizygosity and sex-specific life history differences, which is not possible with XY and ZW systems [214]. They are also interesting candidates for use in experimental evolution, as they are expected to experience a faster response to sex-specific selection compared to diploid systems, since both sexes will experience selection for recombination arrest [228]. It may therefore be possible to gain new insights into the evolution of XY and ZW systems by studying UV systems.

Our survey of the literature revealed much more evidence of effects of Y, W and U and V chromosomes on non-sexual traits than we had initially anticipated. Given the fact that such effects were in many cases unexpected and unlooked for (e.g. [183]), it seems likely that we are currently only seeing the tip of the iceberg, and that many more examples of the genetic potential of NRSCs are waiting to be discovered.

## Data Availability

Not applicable.
